# Differential effects of acute and chronic antagonist and an irreversible antagonist treatment on cocaine self-administration behavior in rats

**DOI:** 10.1038/s41598-022-12798-x

**Published:** 2022-05-24

**Authors:** Hanna N. Wetzel, Vladimir L. Tsibulsky, Andrew B. Norman

**Affiliations:** 1 Department of Pharmacology and Systems Physiology, University of Cincinnati, College of Medicine, 231 Albert Sabin Way, Cincinnati, OH 45267-0575 USA; 2 Present Address: Department of Biology, Xavier University, 3800 Victory Parkway, Cincinnati, OH 45207 USA

**Keywords:** Addiction, Addiction

## Abstract

According to pharmacological theory, the magnitude of an agonist-induced response is related to the number of receptors occupied. If there is a receptor reserve, when the number of receptors is altered the fractional occupancy required to maintain this set number of receptors will change. Therefore, any change in dopamine receptor number will result in a change in the concentration of cocaine required to induce the satiety response. Rats that self-administered cocaine were treated with the irreversible monoamine receptor antagonist, EEDQ, or were infused continuously for 14 days with the D_1_-like antagonist, SCH23390, treatments known to decrease or increase, respectively, the number of dopamine receptors with a concomitant decrease or increase in response to dopaminergic agonists. The rate of cocaine maintained self-administration increased or decreased in rats treated with EEDQ or withdrawn from chronic SCH23390 infusion, respectively. After EEDQ treatment, the effect ratio of a single dose of SCH23390 or eticlopride were unchanged, indicating that the same SCH23390- and eticlopride-sensitive receptor populations (presumably dopamine) mediated the accelerated cocaine self-administration. Changing the receptor reserve is a key determinant of the rate of cocaine self-administration because the resulting increased or decreased concentration of cocaine results in an accelerated or decelerated rate of cocaine elimination as dictated by first-order kinetics.

## Introduction

Despite decades of research that have identified dopaminergic neurotransmission as a primary mediator of the actions of cocaine^1,2^, clinical trials of numerous competitive dopamine receptor antagonists have all failed to provide sufficient evidence of clinical efficacy for the treatment of cocaine use disorders^3,4^. All of these clinical trials used standard antagonist dosing regimens that were designed to achieve a quasi-steady state for several weeks. In animal studies, such long-term treatments typically result in supersensitive dopaminergic neurotransmission characterized by an increase in the behavioral response to direct and indirect dopamine receptor agonists and a concomitant increase in the number of dopamine receptors^5^.

Drug self-administration by animals is a commonly used model of human substance use disorder. Maintained self-administration of the indirect dopamine agonist cocaine can be explained by a pharmacokinetic/pharmacodynamic (PK/PD) model where the time between successive self-administrations is determined by the time for the cocaine concentration to decline back to the satiety threshold^6^. The satiety threshold is defined as the maximal concentration (level) of cocaine at which the probability of self-administration approximates one and above which the probability of self-administration is low^6^. While the satiety threshold model was established using an FR1 schedule, it has also been used to describe the progressive ratio schedule^7^. This model assumes constant PK elimination parameters for cocaine, resulting in a constant relationship between the unit of self-administered cocaine and the levels of cocaine produced in the rats. This allows us to predict cocaine levels at satiety threshold and to make specific predictions about changes in cocaine levels as a function of dopamine receptor antagonism. Specifically, both D_1_-like^8,9^ and D_2_-like^2,10,11^ competitive dopamine receptor antagonists accelerate cocaine self-administration behavior in rats. According to the theory of competitive antagonism, receptor antagonists increase the agonist concentration required to produce a defined magnitude of the response^12^. This equiactive agonist concentration is assumed to correspond to the occupancy of a specific *number* of receptors. This pharmacological theory of competitive antagonism is applicable to cocaine maintained self-administration behavior in rats. In this case the satiety threshold (*D*_*ST*_) is assumed to represent an equiactive agonist concentration that should be increased in the presence of a competitive antagonist^13^. Consequently, it was proposed that the decrease in inter-injection interval (*T*) of a unit dose of cocaine is caused by a PK/PD interaction where the absolute rate of cocaine elimination is faster at higher concentrations, as dictated by first-order kinetics, so that cocaine levels decline more rapidly to the elevated satiety threshold^13^. Although there is a substantial literature on the acute effects of competitive dopamine receptor antagonists in this model, the effects of protracted treatments with these compounds has been neglected, despite the clear clinical significance. To our knowledge only one study of chronic treatment with a dopamine receptor antagonist on cocaine self-administration has been reported, which studied the selective D_1_-like competitive antagonist, SCH23390, in non-human primates^14^. It was reported that in some monkeys there were decreases in the rate of responding to a moderate dose of cocaine after the withdrawal from chronic SCH23390 treatment, though the significance of this change was not emphasized.

Typically, not all of the receptors in a population need to be occupied by an agonist in order to induce a maximum response, and these represent a receptor reserve. This receptor reserve (sometimes referred to as spare receptors) is the mechanism by which the sensitivity to an agonist is increased by reducing the concentration of agonist required to occupy the necessary number of receptors to induce any defined magnitude of response^15^. The aim of this study was to determine the effects of treatments that have been shown to increase the dopamine receptor populations and/or increase sensitivity to dopamine agonists, or to decrease the dopamine receptor population in order to determine the relationship between estimated dopamine receptor activity on the rates of cocaine self-administration in rats. Receptor inactivation was achieved using the irreversible receptor antagonist N-ethoxycarbonyl-2-ethoxy-1,2-dihydroquinoline (EEDQ), which has been shown to inactivate dopamine receptors in the brain in vivo without affecting the number of dopamine transporters^16,17^. EEDQ has been shown to deactivate both D_1_-like^16^ and D_2_-like^18^ dopamine receptors. Receptor supersensitivity was achieved through a chronic infusion of the D_1_-like competitive antagonist, SCH23390. It has been shown that daily injections of SCH23390 (0.5 mg/kg/day) for 21 days resulted in a significant up-regulation of D_1_ receptor binding activity in the rat brain^19–21^. Chronic administration of SCH23390 has also been shown to cause supersensitivity to dopamine receptor agonists demonstrated in electrophysiological and behavioral studies^21,22^.

## Methods

### Animals

Male Sprague–Dawley rats between 200 and 500 g during the course of the study were purchased from Harlan Laboratories (Indianapolis, IN). Rats were housed individually on a 14/10-h light/dark cycle with unrestricted access to food and water. All studies were conducted in accordance with the National Institutes of Health *Guide for the Care and Use of Laboratory Animals* and under a protocol approved by the Institutional Animal Care and Use Committee at the University of Cincinnati, and reported in accordance with ARRIVE guidelines.

### Self-administration training

Rats were implanted with indwelling catheters into the right jugular vein under isoflurane anesthesia, followed by the left jugular and femoral veins which were catheterized as needed throughout the study. Bupranex (0.03 mg/rat s.c.) was administered for pain relief and gentamycin (25 mg/animal s.c.) was used to prevent infection following surgery. Detailed protocols for cocaine self-administration can be found in Tsibulsky and Norman^23^. In brief, beginning 5 days after surgery, rats were trained to self-administer cocaine HCl. Rats were weighed daily immediately prior to each self-administration session. Animals were placed in isolated chambers containing an active and inactive lever. During training, a unit dose of 3 μmol/kg was delivered on a fixed-ratio 1 (FR1) schedule with a timeout period equal to the time of the injection or 5 s, whichever is longer. A cue-light was illuminated for the duration of timeout. Rats had access to cocaine for 3–4 h a day, 5 days a week. Training was considered complete when inter-injection intervals did not deviate significantly and systematically from the mean for three consecutive sessions.

### Self-administration procedures

The self-administration protocol used here was identical to that used previously in this laboratory^24^. In short, session began between 8:00 and 10:00 a.m., 6 days a week (Monday through Saturday). First, rats were placed in the chamber, and a cue-light associated with cocaine injection was illuminated after every active lever press and at variable intervals of 100–600 s until no lever presses occurred for 30 min. This was done to eliminate the interference of cue-induced lever pressing with the measurement of cocaine-induced pressing. Once lever-pressing was extinguished, programmed non-contingent injections of cocaine were given every two minutes at escalating doses in order to gradually raise the concentration of cocaine in the rat. When the rat pressed the active lever 5 times with each interval of less than 1 min, it was assumed that self-administration had been reinstated. If the calculated cocaine concentrations reached 10.0 μmol/kg it was assumed that the animal could not be safely primed and the session was terminated. If the animal was primed, it was allowed to receive 20 injections of a 3 μmol/kg unit dose. After that, the lever was deactivated and animals were left in their chambers until 30 min had passed since their last lever press, at which time animals were returned to their home cages.

### Estimations of cocaine level in the body

Cocaine level in the animal was calculated during each self-administration session. Complete protocols for the calculation of cocaine level in the rat’s bodies were can be found in Tsibulsky and Norman^23^. Briefly, the cocaine level in the body was calculated every second using a one-compartment pharmacokinetic model and assuming 500 s elimination half-life.

### The effects of SCH23390 on cocaine self-administration

The baseline values of the inter-injection intervals at 0.3 and 3.0 μmol/kg unit doses were collected for at least 3 weeks prior to the Alzet osmotic pump implantations. Alzet pumps (0.5 µl/h for 14 days, model 2002) were implanted subcutaneously into the back, slightly posterior to the scapulae in the rats under isoflurane anesthesia. These pumps were filled with SCH23390 solutions in saline at three concentrations producing three rates of drug infusion: 26.7 ± 1.7 nmol/kg/h (n = 3), 52.5 ± 3.6 nmol/kg/h (n = 4) and 69.6 ± 2.1 nmol/kg/h (n = 9). For comparison with other published results, the infusion rates were 208, 408 and 541 μg/kg/day, respectively.

Self-administration sessions were conducted on Day 1, 3, and 10 after the pumps were implanted. 14 days after implantation, pumps were extracted under isoflurane anesthesia. Daily self-administration sessions were resumed 1 day after pump extractions and continued for at least 4 weeks.

### The effects of EEDQ on cocaine self-administration

Rats were primed using the procedure stated above. After rats had reinstated self-administration, they were allowed to self-administer until stable baseline was established for about 1 h (10–13 self-injections). Immediately following an injection, the rats were removed from the chamber, detached from the syringe, and injected with EEDQ (1 mg/kg in 10% ethanol in saline i.v.) or vehicle (10% ethanol in saline). The animal was immediately reattached to the syringe with cocaine, and put back into the chamber. The rats were allowed to continue self-administration for about 1 h, or if the animal ceased self-administration (determined by no lever-pressing for at least 30 min) they were removed from the chamber and returned to home cages. Self-administration sessions were conducted at 8, 16, 24, 32, 40, 48, 56, 68, 80, 92 and 96 h after injection and then every 24 h until inter-injection intervals returned to baseline. Inter-injection intervals and calculated cocaine levels at the time of lever press during maintenance were recorded during every session.

### Determination of the potency of SCH23390 and eticlopride before and after EEDQ treatment

To determine the continued involvement of D_1_-like and D_2_-like dopamine receptors in the mediation of satiety threshold following EEDQ treatment, the potencies of SCH23390 (D_1_-like selective competitive antagonist) and eticlopride (D_2_-like selective competitive antagonist) were measured. Another group of trained rats were allowed to self-administer until stable baseline was established for about 1 h. Immediately following a cocaine injection, the rat was removed from the chamber and injected with either eticlopride or SCH23390 (each at 20 nmol/kg i.v.) via the same i.v. catheter. Rats were reattached to the cocaine-containing syringe, placed back in the chamber and self-administration resumed. Rats were allowed to continue self-administration for 3–4 h until inter-injection intervals approached baseline. Injections of each antagonist were repeated 3–4 times for each rat, with at least 2 days between sessions.

Following these baseline experiments, the same rats were injected with EEDQ (1 mg/kg). Beginning 4 h after EEDQ injection, attempts to prime the rats were made every 8 h. As soon as self-administration was reinstated, they were allowed to self-administer for approximately 1 h to establish the new stable baseline intervals. Animals were removed from the chamber, detached from the syringe, and injected with either eticlopride or SCH23390 (20 nmol/kg i.v.) as done prior to EEDQ treatment. Rats were reattached to the syringe and placed back in the chambers and allowed to continue self-administration until inter-injection intervals returned to the elevated baseline. On five occasions, competitive antagonist treatment abolished self-administration behavior or the elevated cocaine concentrations induced seizures. In these cases, the antagonist injections were repeated every 24 h until reliable self-administration following treatment was achieved. The ratio of the highest level of cocaine at the time of lever-presses after antagonist injection to the mean baseline levels during the same session in the same rat was calculated. All injections of dopamine antagonists were given within 4 days after EEDQ administration.

### Drugs

(−)-Cocaine HCl was provided by the Research Triangle Institute (Chapel Hill, NC) under the National Institute on Drug Abuse drug supply program. EEDQ, SCH23390 HCl and S(−)-Eticlopride HCl were purchased from Sigma-Aldrich, St. Louis, MO. Cocaine was dissolved in saline at the concentration of 40 µmol/ml. EEDQ was dissolved in 95% ethanol at the concentration of 2.0 mg/ml immediately or not more than 3 days before the injection. Stock solution of SCH23390 and Eticlopride were dissolved in 95% ethanol at the concentration of 20 μmol/ml, were stored at − 20 °C. Solutions were further diluted before animal injections for a maximum ethanol concentration of 10%. The dose of ethanol injected along with eticlopride or SCH23390 was 0.02 mg/kg and did not affect cocaine self-administration behavior^26^.

### Data analysis and statistics

Baseline inter-injection interval values within sessions were typically log-normally distributed. Therefore, all statistical analyses of these two parameters were performed using their logarithmic values. Inter-injection intervals were averaged for each session. Baseline trends were determined for at least 3 weeks prior the vehicle, EEDQ, or SCH23390 injections. Baseline values of mean inter-injection intervals were extrapolated for each rat using linear regression analysis. Following treatment, the values of mean inter-injection intervals were compared with respective baseline values expected on the same day using a paired t-test, as previously reported^24^. Nonlinear regression analyses of the recovery of inter-injection intervals and the estimation of treatment effect half-lives were calculated according to a mono-exponential equation in each individual rat.

The effects of competitive dopamine receptor antagonists on inter-injection intervals before and after EEDQ injections were statistically assessed using a paired t-test comparing mean ratios before EEDQ and ratios in the session immediately following EEDQ. Graphic and statistical analyses were conducted using SigmaPlot (Systat Software Inc., San Jose, CA). Multiple comparison correction was performed according to the False Discovery Rate (FDR) method^25^. The significance level was set at *p* = 0.05.

### Significance statement

Irreversible and competitive antagonist treatments that reduce or increase dopamine receptor number in the brain accelerate or decelerate, respectively, cocaine self-administration in rats. While the acute effect of competitive dopamine receptor antagonists is to accelerate self-administration behavior, withdrawal from chronic dopamine receptor antagonist treatment has the opposite effect. Dopamine receptor concentrations vary in a number of situations, including substance use disorders, and as a result of the natural aging process. Changes in receptor numbers in individual humans could influence cocaine use.

## Results

### Effect of SCH23390 infusion on Inter-Injection Intervals

**Figure 1:**
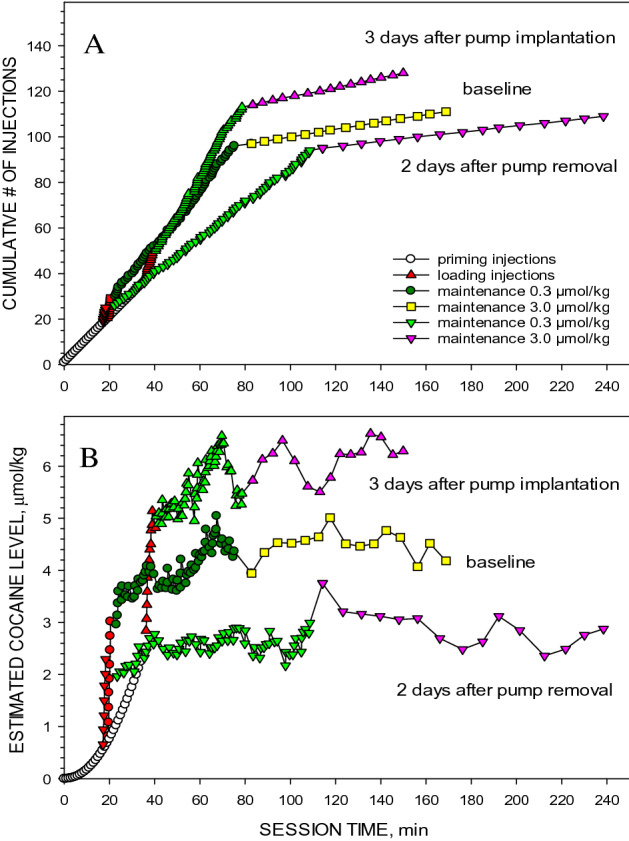
The effect of a 14-day infusion of SCH23390 on inter-injection intervals (Panel **A**) and calculated cocaine levels (Panel **B**) in representative sessions from the same rat before, during and after implantation of the Alzet pumps. Open circles represent programed response-independent (non-contingent) injections. The red symbols represent the loading injections. The green symbols represent injections in the first half of the maintenance phase at the 0.3 µmol/kg, while the pink symbols represent injections in the second half of sessions at the 3.0 µmol/kg unit dose.

Representative cocaine self-administration sessions from the same rat before, during and after a 2-week infusion of SCH23390 are shown in Fig. 1. In all sessions, after self-administration was reinstated by the programmed injections of cocaine there was a brief loading period characterized by very short inter-injection intervals (Fig. 1A). Subsequently, the self-administration of 0.3 µmol/kg of cocaine was characterized by short and regular inter-injection intervals. When the unit dose of cocaine increased tenfold, there was an abrupt increase in inter-injection intervals, and these intervals were also regular. This same pattern was seen in all three sessions. However, after implantation of the Alzet pumps the inter-injection intervals were significantly shorter compared with the baselines at both unit doses. After the withdrawal of the constant SCH23390 infusion, the inter-injection intervals at each unit dose were considerably longer than those observed before the beginning of the antagonist infusion.

In both sessions, despite the large increase in inter-injection intervals when the unit dose was increased tenfold, there was little change in the calculated cocaine concentrations at the time of each lever press (Fig. 1B). However, after implantation of the Alzet pumps the minimal maintained cocaine level increased significantly at both unit doses. After the withdrawal of the constant SCH23390 infusion, the calculated cocaine level at the time of each lever press at both unit doses were considerably lower than that observed before and during the antagonist infusion.

**Figure 2:**
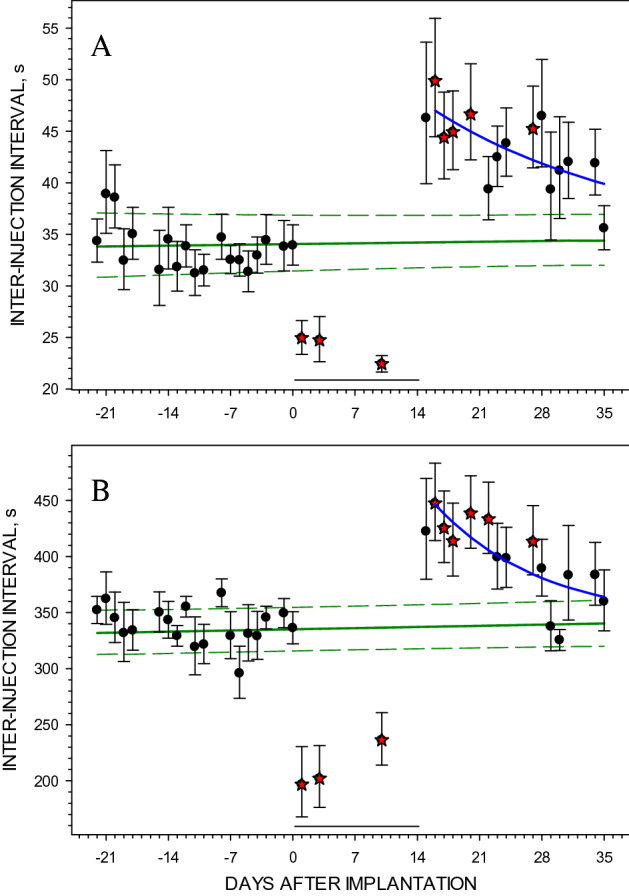
The effect of a 14-day infusion of SCH23390 on the rate of cocaine self-administration. The panels show the percent of baseline inter-injection intervals at a cocaine unit dose of 0.3 µmol/kg (Panel **A**) and 3.0 µmol/kg (Panel **B**) before, during and after SCH23390 infusion. The green straight lines represent mean interpolated baseline with ± 95% CI from 9 rats indicated by the dashed black lines. Symbols represent mean ± SEM inter-injection intervals, which are not different (closed circles) or statistically different (paired t-test, *p* < 0.05, red stars) from the projected baseline. The black horizontal lines on the bottom of each panel show the infusion days. The blue curved lines represent the best fit of a mono-exponential regression approximation to the post-infusion inter-injection intervals. Effect decay half-lives were 13.6 days at 0.3 µmol/kg and 8.1 days at 3.0 µmol/kg.

During the infusion of SCH23390, the mean inter-injection intervals were significantly decreased at both cocaine unit doses (Fig. 2A,B) across the 14 days of infusion. Analyses showed significant effects of treatment on the inter-injection intervals at 0.3 μmol/kg (the one-way ANOVA F_1,15_ = 136.3, *p* < 0.001) and at 3.0 μmol/kg unit dose (F_1,15_ = 138.95, *p* < 0.001). The acceleration of cocaine self-administration was similar to the acute effects of a single dose of SCH23390^8,26^. After removal of the Alzet pumps the mean inter-injection intervals increased to 49.9 s (increase over the baseline by 49.9% at 0.3 μmol/kg) and to 447.4 s (by 32.6% at 3.0 μmol/kg), then gradually returned to the baseline levels over the next 2 weeks. The one-way ANOVA showed a significant effect of withdrawal on the inter-injection intervals both at 0.3 μmol/kg (the one-way ANOVA, F_1,17_ = 264.8, *p* < 0.001) and 3.0 μmol/kg (F_1,17_ = 91.86, *p* < 0.001). The half-life of the recovery rate was in the range between 8 and 14 days (Fig. 2).

### The effects of EEDQ on maintenance of self-administration

**Figure 3:**
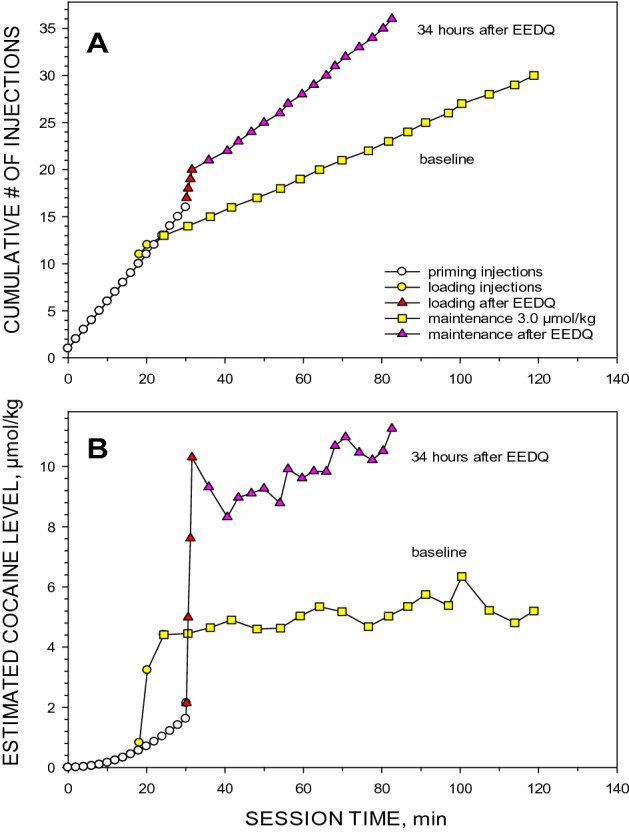
The effect of EEDQ on cocaine self-administration. The panels are two representative sessions from the same rat before (yellow squares) and 34 h after (pink triangles) EEDQ injection. Panel (**A**) shows the cumulative number of injections over session time, while Panel (**B**) shows the calculated cocaine level in the rat at the time of each injection.

**Figure 4:**
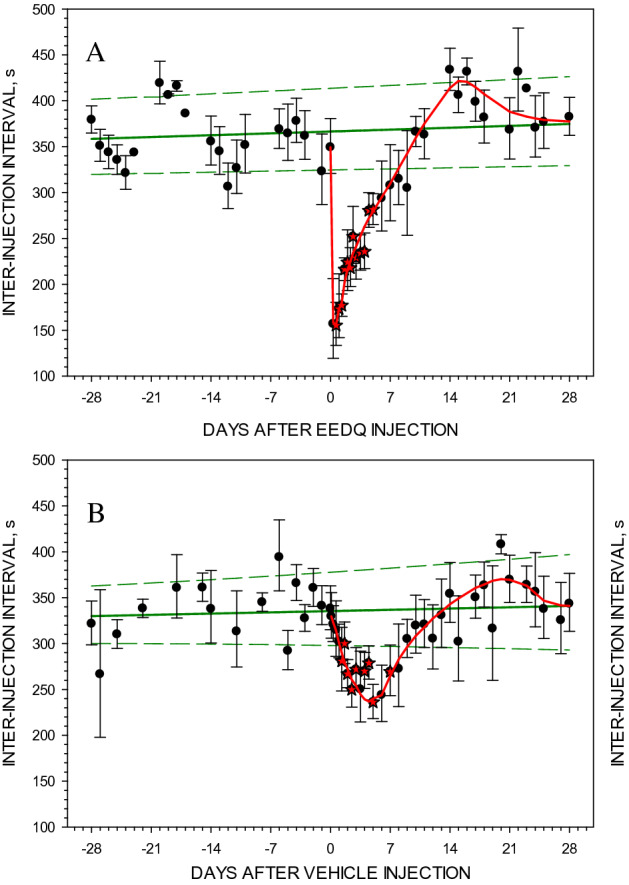
Effect of EEDQ (Panel **A**) or a vehicle (Panel **B**) injection on inter-injection intervals at a cocaine unit dose of 3.0 μmol/kg. EEDQ was injected 24 or 32 days after vehicle injection. Solid straight lines (green) represent linear regression of geometric means ± 95% confidence intervals of inter-injection baseline intervals from 7 rats. Each data point represents the mean ± SEM from 7 rats. Solid curved lines (red) represent the best approximation to the vehicle/frequent access effect and to EEDQ effect drawn based on visual analysis.

Due to the lack of difference in the effect on intervals at the 0.3 and 3.0 µmol/kg doses in the previous experiment with chronic SCH23390, only the 3.0 µmol/kg unit dose was used in the following experiments. This shortened the self-administration session, thus minimizing exposure to cocaine while maximizing data collection. After EEDQ exposure rats were able to maintain stable intervals between cocaine injections (Fig. 3). At the peak of the effect, 24 h after EEDQ injection, inter-injection intervals were 52.8% shorter (the mean 173.3 s, with the SD range of 116.4–258.2 s) than baseline values in all rats (367.0 s, 324.9–414.2 s). The one-way ANOVA showed the significant effect of EEDQ (F_1,21_ = 108.094, *p* < 0.001). Intervals gradually returned to the baseline levels within 7–10 days (Fig. 4A). This recovery process was approximated by the equation for mono-exponential growth to the maximum. The average recovery half-life was 2.9 ± 0.3 days.

### The effects of a frequent access protocol on self-administration behavior

A group of six animals (three of them were the same rats as in EEDQ group) was injected with a vehicle control, but exposed to the same frequent access self-administration schedule as the EEDQ treated animals. Initially, the frequent access protocol caused a significant decrease in inter-injection intervals. However, this effect was variable between rats (Fig. 4B). The one-way ANOVA showed that the frequent access protocol significantly decreased inter-injection intervals (F_1,18_ = 16.78, *p* < 0.001). The effect reached the peak of − 28.7% on Day 5 after the beginning of the frequent access protocol. The recovery started immediately after the interval between sessions returned to the standard 24 h and was complete in 5 more days. The average recovery half-life was 12.3 ± 4.6 days.

### The magnitude of response to SCH23390 and eticlopride after EEDQ treatment

Following the injection of the competitive antagonists eticlopride or SCH23390, the inter-injection intervals were shorter (data not shown). This acceleration of cocaine self-administration behavior is consistent with previously published observations^9,11,13^. The rate of self-administration plateaued in approximately 25–30 min for both competitive antagonists, and then gradually returned towards the pre-injection rate. The mean baseline inter-injection intervals for sessions before eticlopride injection was 341.4 ± 20.2 s and the plateau interval was 191.6 ± 8.7 s. The mean baseline inter-injection intervals for sessions before SCH23390 injection was 395.9 ± 17.4 s and the plateau interval was 181.1 ± 9.7 s. In rats administered EEDQ, the competitive antagonists also produced an acceleration of self-administration behavior, in addition to that produced by EEDQ, with the same pattern of a plateau and subsequent return to the pre-injection levels. The time-course of the competitive antagonist effects was similar to that observed in the animals when not treated with EEDQ. The mean pre-eticlopride inter-injection interval was 230.0 ± 21.2 s and the plateau interval was 119.0 ± 8.2 s. The mean pre-SCH23390 inter-injection interval was 291.4 ± 23.5 s and the plateau interval was 127.6 ± 9.8 s. The ratios of the plateau rates compared to pre-injection rates are presented in Table 1. There were no significant differences in these ratios for each competitive antagonist in rats administered EEDQ or not administered EEDQ.

**Table 1:** The effect of an injection of EEDQ on the potency of eticlopride and SCH23390 to elevate the rate of cocaine self-administration and the satiety threshold (Dst).

Competitive antagonists	Ratio before EEDQ		Ratio after EEDQ		n
	Rate	D_ST_	Rate	D_ST_	
Eticlopride (20 nmol/kg)	1.80 ± 0.07	2.08 ± 0.07	1.93 ± 0.11	2.12 ± 0.13	5
SCH23390 (20 nmol/kg)	2.20 ± 0.11	2.84 ± 0.13	2.31 ± 0.20	3.12 ± 0.22	4

The ratio of the rate of cocaine maintained self-administration and the peak satiety threshold after injection of competitive antagonist compared to values prior to injection. Ratios were not significantly different before or after EEDQ treatment for either eticlopride or SCH23390.

**Figure 5:**
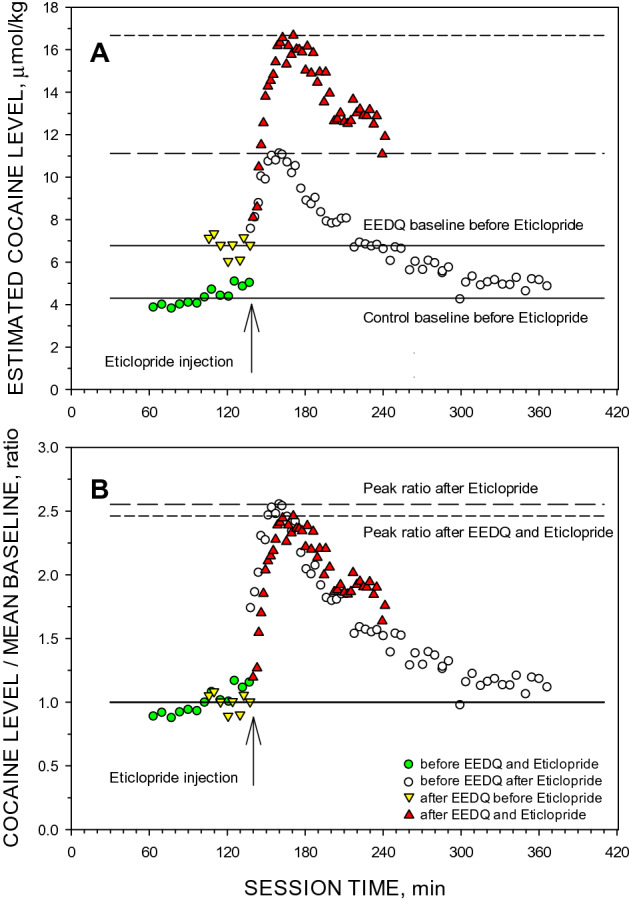
The effect of an EEDQ injection on the potency of eticlopride to elevate the cocaine satiety threshold during cocaine self-administration. The panels show two representative sessions of cocaine self-administration in the same animal before and after EEDQ injection. Panel (**A**) shows the calculated cocaine concentration over time. Panel (**B**) shows ratios of cocaine levels at the time of injection to the mean of corresponding baseline levels. Circles represent concentrations before (green) and after (open circles) eticlopride injection in the absence of EEDQ. Triangles represent cocaine concentrations before (yellow) and after (red) eticlopride injection in the presence of EEDQ. Solid lines show mean baselines cocaine levels one day before EEDQ injection (4.3 μmol/kg) and 40 h after EEDQ injection (6.8 μmol/kg). Dash lines show the maximal cocaine level after eticlopride (20 nmol/kg i.v.) injection on the day before (11.1 μmol/kg, long-dash) and after EEDQ injection (16.7 μmol/kg, short-dash). The ratios of the estimated peak concentration to the corresponding baselines were 2.6 and 2.5, respectively.

The ratios of the peak satiety threshold to baseline satiety threshold is an indication of the potency of dopamine antagonists. A representative session is shown in Fig. 5. The ratio between baseline and the peak effect was measured before and after EEDQ injection. This was used as an indicator of the antagonist potency when the total number of dopamine receptors had been significantly reduced. EEDQ significantly increased the satiety threshold. Despite a large increase in baseline satiety threshold from 4.6 ± 0.2 μmol/kg to 8.1 ± 0.9 μmol/kg on the first successful priming session after EEDQ injection, there was no significant difference in the ratio of baseline to the peak effect after SCH23390 or eticlopride injection (Table 1).

## Discussion

### Acute reversible antagonist treatment

The acceleration of cocaine self-administration after pre-session systemic injections of selective D1 and D2 dopamine receptor antagonists are well established^8,27^. The acceleration, plateau and subsequent slowing of cocaine self-administration after a single i.v. injection of reversible dopamine receptor antagonists during the maintenance phase of a session were observed in this study^9^. The cocaine level at the time of lever press during cocaine maintained self-administration represents the satiety threshold and is assumed to be an equiactive agonist concentration^13^. Competitive antagonists increase the equiactive agonist concentration, and the ratio of this concentration before and after antagonist treatment is a measure of antagonist potency^28^. Therefore, the ratio of the cocaine satiety threshold before and after eticlopride or SCH23390 is a measure of their potencies. A dose of antagonist that produces a twofold increase in the equiactive agonist concentration represents the Kdose, which is approximately 20 nmol/kg for both eticlopride and SCH23390^9^.

### Chronic reversible antagonist treatment and withdrawal

During the continuous infusion of SCH23390, cocaine self-administration was accelerated with a decrease in intervals at a similar magnitude observed after a single injection of SCH23390. This indicates that SCH23390 was actively antagonizing dopamine receptors throughout the infusion.

**Figure 6:**
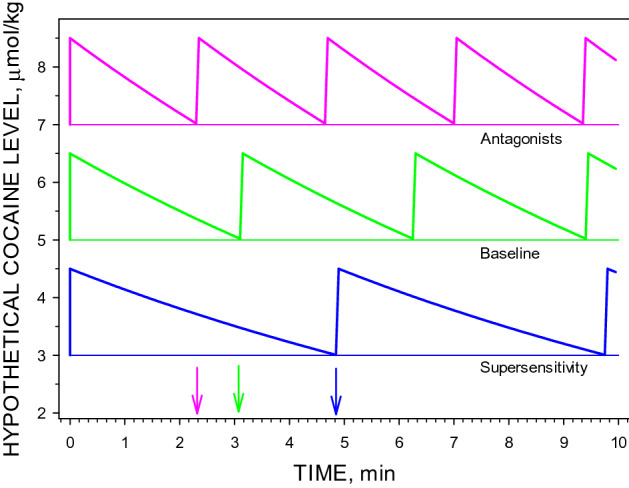
A pharmacokinetic/pharmacodynamic (PK/PD) interaction model. To summarize these results, a model was generated of the effects of irreversible antagonism and supersensitivity on self-administration behavior of the same cocaine unit dose, and assuming that the first-order elimination rate constant of cocaine was unaltered by the treatments. Compared to baseline (green line), supersensitivity of receptors (blue line) results in a decreased satiety threshold. At the lower concentrations, the rate of elimination of cocaine is lower, as dictated by first-order kinetics, and it takes longer for the concentration to decline back to the satiety threshold, resulting in a longer inter-injection interval. Receptor antagonism by both reversible and irreversible antagonists (magenta line) results in an increased satiety threshold. At the higher concentrations, the rate of elimination of cocaine is faster and it takes a shorter time for the concentration to decline back to the elevated satiety threshold resulting on a shorter inter-injection interval. The horizontal lines represent the satiety threshold under each condition. The arrows represent the inter-injection interval duration for each condition.

Withdrawal from chronic treatment with SCH23390 reveals an upregulation of D_1_-like receptors^20^ and produces an increased behavioral response to dopamine receptor agonists^29^. In the present study, the effects of supersensitive dopamine systems are characterized by a marked decrease in the rate of cocaine self-administration on Days 2–7 after discontinuing the chronic antagonist infusion (Fig. 2), which is consistent with the report of the effects of chronic SCH23390 on cocaine self-administration in monkeys^14^. This deceleration of self-administration behavior resulting from a supersensitive system is opposite to the acceleration induced by a single dose of SCH23390. We have previously proposed that the SCH2330-induced acceleration of cocaine self-administration behavior is the result of a PK/PD interaction where an increase in the satiety threshold results in an increase in the rate of elimination of cocaine^13^. Similarly, a supersensitive dopamine system would result in a decrease in the cocaine satiety threshold. Consequently, the rate of cocaine elimination would be slower at the lower concentrations, as dictated by first-order kinetics, and it would take longer for cocaine concentrations after injection to fall to the lowered satiety threshold. Figure 6 illustrates this model.

The magnitude of the deceleration of cocaine self-administration behavior was substantial and the effect was measurable for more than a week before returning to baseline. Therefore, the observed effect on cocaine self-administration behavior during and after chronic antagonist treatment is opposite when the antagonist concentration declines and uncovers a supersensitive dopamine system.

The supersensitivity of agonist-induced responses after chronic antagonist treatments is typically assumed to be due to the observed increase in the number of receptors^5^. Indeed, the treatment of rats with adenovirus that carried the D_2_ receptor gene to upregulate D_2_ dopamine receptors in the nucleus accumbens resulted in a significant decrease (75%) in cocaine consumption. The duration of this effect corresponded to the time needed for the number of D_2_ receptors to return to baseline^30^. It is likely that the increase in receptor number results in an increased receptor reserve, which enhances the sensitivity of the system to receptor agonists by reducing the concentration of agonist required to induce a defined magnitude of response^15^.

### Acute irreversible antagonist treatment

The injection of a single dose of EEDQ resulted in immediate acceleration of cocaine self-administration. This effect remained long after the EEDQ would have cleared from the rat (Fig. 4A), for which the longest estimates are about 24 h, consistent with the irreversible antagonism of the receptors underlying this behavior^31^. If so, the observed acceleration of self-administration behavior was due to the decreased total number of receptors. The time course of recovery of the rate of self-administration behavior is consistent with the rate of recovery of both D_1_- and D_2_-like dopamine receptor populations in rat striatum after a single treatment with EEDQ^16,18^. This implies that the satiety response requires only a relatively low number of dopamine receptors. This indicates that there is a substantial receptor reserve in the systems underlying cocaine maintained self-administration behavior. If the effect of EEDQ is similar to that observed in previous studies^16,18^, then the satiety response may be observed when only approximately 20% of D_1_- and D_2_-like dopamine receptors are present. It is possible that receptor transduction efficiency is also changed after EEDQ treatment, which might account for the rapid reappearance of cocaine self-administration behavior, typically within a day after EEDQ. The observed acceleration of cocaine self-administration after EEDQ treatment is consistent with the report that in mutant mice lacking D_2_ receptors the rate of cocaine self-administration was accelerated^32^.

In the case of the EEDQ-induced acceleration of cocaine self-administration, the reduction of the number of receptors reduced the receptor reserve, so a higher concentration of agonist is required in order to occupy the fixed number of receptors required to produce a particular magnitude of response. It is a fundamental principle of pharmacology that a set number of receptors that are occupied by an agonist will induce a particular magnitude of response. Therefore, the number of receptors occupied by an agonist at the satiety threshold should also be constant. This number may be constant under any situation, whether there is a normal, a depleted or an increased receptor population. If so, the mechanistic definition of the satiety threshold would be the minimum number of receptors required to induce the satiety response. In contrast to this mechanistic definition of the satiety threshold, the operational definition of the satiety threshold was based on the minimum dose of cocaine required to produce a cocaine concentration that induced the satiety response^6^.

### More frequent access to cocaine

The increase in the rate of cocaine self-administration observed in the control (vehicle treated) rats in the days following the vehicle injection is likely due to the increased exposure to self-administered cocaine. During this time, the sessions were run every 8 h, rather than daily, and the effect is similar to the reported escalation of cocaine intake observed for ten days under a frequent access daily regimen of self-administration^33^. For the first 3 days after injection of either vehicle or EEDQ, the total duration of three self-administration sessions was about 6 h. It has been demonstrated that the single long access session of 6 h also results in significant decrease of inter-injection intervals at a wide range of cocaine unit doses^34^. It is possible that this phenomenon can be explained by the development of tolerance to cocaine with more frequent access. More cocaine would be required to induce the same magnitude of response, and would be consistent with a down regulation of the number of dopamine receptors.

### Change in fractional occupancy

If the number of occupied receptors required to induce the satiety response is constant, a change in the total number of receptors results in a change only in the receptor reserve and, therefore, in the fraction of the total receptor population required to induce the satiety response. This fraction is increased by EEDQ because the total number of receptors is decreased, and decreased by chronic SCH23390 because the total number of receptors is increased. Fractional occupancy by a ligand is dependent on a ligand’s affinity and concentration. Assuming unchanged affinity, the increased fractional occupancy will require a higher concentration of a ligand. Consequently, if it is assumed that the number of occupied receptors required to induce the satiety response is constant, then fractional occupancy of the remaining receptor population after EEDQ must be increased. At the elevated satiety threshold concentration, the cocaine elimination rate is increased as dictated by first-order elimination kinetics. As a result, the cocaine concentration produced by a unit dose of cocaine decreases more rapidly to the elevated satiety threshold concentration, thereby shortening the inter-injection interval, similar to the effect of a single injection of a competitive antagonist. This explanation is illustrated in Fig. 6.

The response to irreversible antagonism of dopamine receptors was similar to that produced after acute treatment with the competitive D_1_-like receptor antagonist, SCH23390 (Fig. 2 and Table 1), and the competitive D_2_-like receptor antagonist eticlopride (Fig. 5). Despite the similar acceleration of self-administration behavior, these two classes of antagonists have distinct mechanisms of action, with only EEDQ changing the number of available receptors. The SCH23390- and eticlopride-induced acceleration of cocaine self-administration was previously explained by an increase in the cocaine concentration required to induce the same magnitude of a quantal response, corresponding to the satiety threshold^9^. Importantly, it is the change in cocaine concentration that results in the change in intervals in the presence of competitive antagonists, EEDQ, or in a supersensitive system.

It has previously been shown that the potencies of competitive dopamine receptor antagonists can be determined using Schild analysis of the increase in satiety threshold as a function of antagonist dose^9^ Since the cocaine concentration ratio for the same dose of SCH23390 or eticlopride was not altered after a treatment with EEDQ it is concluded that the pharmacology of the receptor populations underlying cocaine self-administration were unaltered. Since EEDQ is not selective for subtypes of dopamine receptors, or for several monoamine receptors, the continued involvement of dopamine receptors following this non-selective receptor knock-down was confirmed by the unchanged satiety threshold ratios, and therefore relative potencies, of the selective D_1_-like (SCH23390) and D_2_-like (eticlopride) competitive antagonists (Fig. 5, Table 1).

## Summary and limitations

There are a few key limitations of this research. First, only male rats were included. The lack of inclusion of female rats is a weakness. Additionally, all experiments were done using an FR1 schedule and animals were primed using non-contingent doses of cocaine. This may limit the translation of this work into humans. Lastly, cocaine levels in the animals were all calculated and not measured.

In summary, treatments that have been shown to produce an increase or a decrease in receptor number result in opposite effects on the rate of cocaine self-administration behavior. At the changed cocaine concentration required to occupy the same number of receptors, the rate of elimination of cocaine is changed according to the law of first-order kinetics. The change in inter-injection interval is a direct consequence of this pharmacokinetic/pharmacodynamic interaction. The role of receptor number (or efficiency/sensitivity) and receptor occupancy play a key role in regulating the rate of cocaine self-administration behavior. These findings could have clinical relevance. Dopamine receptor concentrations vary in a number of situations, including substance use disorders, and as a result of the natural aging process^35^. Changes in receptor numbers in individual humans could influence cocaine use after protracted antagonist treatment.

## Abbreviations

D_U_: Cocaine unit dose
D_ST_: Satiety threshold
EEDQ: N-ethoxycarbonyl-2-ethoxy-1, 2-dihydroquinoline
FR1: Fixed-ratio 1 schedule of cocaine delivery
k: First-order cocaine elimination rate constant
PK/PD: Pharmacokinetic/pharmacodynamic interaction
T: Inter-injection interval
